# Effects of Human RelA Transgene on Murine Macrophage Inflammatory Responses

**DOI:** 10.3390/biomedicines10040757

**Published:** 2022-03-24

**Authors:** Stamatia Papoutsopoulou, Lorna Morris, Andrew Bayliff, Thomas Mair, Hazel England, Massimiliano Stagi, François Bergey, Mohammad Tauqeer Alam, Raheleh Sheibani-Tezerji, Philip Rosenstiel, Werner Müller, Vitor A. P. Martins Dos Santos, Barry J. Campbell

**Affiliations:** 1 Lydia Becker Institute of Immunology and Inflammation, Faculty of Biology, Medicine and Health, University of Manchester, Manchester M13 9PL, UK; hazel.england@manchester.ac.uk (H.E.); werner.muller@manchester.ac.uk (W.M.); 2 Department of Biochemistry and Biotechnology, School of Health Sciences, University of Thessaly, 413 34 Larissa, Greece; 3 LifeGlimmer GmbH, Markelstr. 39A, 12163 Berlin, Germany; morris@lifeglimmer.com (L.M.); francois.bergey@gmail.com (F.B.); vitor.martinsdossantos@wur.nl (V.A.P.M.D.S.); 4 The Henry Wellcome Laboratories of Molecular & Cellular Gastroenterology, Department of Infection Biology & Microbiomes, Institute of Infection Veterinary and Ecological Sciences, University of Liverpool, Liverpool L69 3GE, UK; a.bayliff@liverpool.ac.uk (A.B.); hltmair@liverpool.ac.uk (T.M.); 5 Department of Molecular Physiology and Cell Signalling, Institute of Systems, Molecular and Integrative Biology, University of Liverpool, Liverpool L69 7BE, UK; maxstagi@liverpool.ac.uk; 6 Warwick Medical School, Bioinformatics RTP, University of Warwick, Coventry CV4 7AL, UK; mdtauqeer@gmail.com; 7 Department of Biology, College of Science, United Arab Emirates University, Abu Dhabi P.O. Box 15551, United Arab Emirates; 8 Institute of Clinical Molecular Biology, Christian Albrechts University of Kiel, 6708 WE Kiel, Germany; raheleh.sheibani@lbiad.lbg.ac.at (R.S.-T.); philip.rosenstiel@uksh.de (P.R.); 9 Laboratory of Systems & Synthetic Biology, Wageningen University & Research, P.O. Box 8033, 6700 EJ Wageningen, The Netherlands

**Keywords:** macrophage, NFκB, RelA(p65), inflammation, lipid A, lipopolysaccharide, toll-like receptor, tumour necrosis factor

## Abstract

The NFκB transcription factors are major regulators of innate immune responses, and NFκB signal pathway dysregulation is linked to inflammatory disease. Here, we utilised bone marrow-derived macrophages from the p65-DsRedxp/IκBα-eGFP transgenic strain to study the functional implication of xenogeneic (human) RelA(p65) protein introduced into the mouse genome. Confocal imaging showed that human RelA is expressed in the cells and can translocate to the nucleus following activation of Toll-like receptor 4. RNA sequencing of lipid A-stimulated macrophages, revealed that human RelA impacts on murine gene transcription, affecting both non-NFκB and NFκB target genes, including immediate-early and late response genes, e.g., *Fos* and *Cxcl10*. Validation experiments on NFκB targets revealed markedly reduced mRNA levels, but similar kinetic profiles in transgenic cells compared to wild-type. Enrichment pathway analysis of differentially expressed genes revealed interferon and cytokine signaling were affected. These immune response pathways were also affected in macrophages treated with tumor necrosis factor. Data suggests that the presence of xenogeneic RelA protein likely has inhibitory activity, altering specific transcriptional profiles of key molecules involved in immune responses. It is therefore essential that this information be taken into consideration when designing and interpreting future experiments using this transgenic strain.

## 1. Introduction

The NFκB family of transcription factors is a major regulator of immune responses, but is also involved in multiple other cellular functions, such as growth and development [1,2,3]. The NFκB family consists of five members, p105/p50, RelA(p65), c-Rel, p100/p52 and Rel-B that form dimers [4]. These dimers are held in the cytoplasm in an inactive state by binding to specific inhibitory proteins, the inhibitors of NFκB (IκB family) [5]. Upon stimulation, the IκB proteins are phosphorylated, ubiquitinated and consequently degraded, leading to translocation of the NFκB dimers into the nucleus where they activate gene transcription [6,7,8].

The amplitude of the NFκB response is tightly regulated and can be affected at the receptor level, as has been shown in studies focused on human T-lymphocytes [9] and macrophages [10]. Also, experiments using murine lymphocytes have revealed that different thresholds are required to activate the NFκB pathway and the apoptotic pathway downstream of wild-type versus heterozygous CD95 receptor activation [11]. Numerous studies have revealed how important integrity of the primary DNA structure is, as mutations can affect the normal function of NFκB proteins: Polymorphisms of genes encoding NFκB subunits have been correlated with dysregulated DNA binding activity [12] across a wide spectrum of diseases, such as Huntington’s disease [13], inflammatory bowel diseases [14,15] and various cancers [16]. A recent study has revealed that haplo-insufficiency in *RELA* results in autosomal-dominant chronic mucocutaneous ulceration in humans [17]. Using NFκB transgenic mice, it has been shown also that genetic manipulation of the p50 subunit can rescue, to a variable extent, target gene expression in macrophages and B-lymphocytes from *Nfkb1*^SSAA/SSAA^ mice [18,19]. These studies highlight that the NFκB subunit allele dose is important for the physiological activation of the NFκB pathway in a cell-specific and target-specific manner. Recently, a novel transgenic mouse expressing human p65 tagged with *Discosoma* red fluorescent protein (DsRedxp) was reported, with confocal microscopy used to monitor human RelA(p65) during the circadian cycle in murine intervertebral disc explant cultures [20], and in cytokine activated bone marrow-derived macrophages (BMDMs) [10]. We also utilised this same strain to generate murine intestinal crypt stem-cell derived 3D organoid cultures expressing p65-DsRedxp, to study the effect of the macrolide antibiotic clarithromycin on p65 nucleocytoplasmic shuttling [21]. To understand further cell-specific pathways in this mouse, we have examined the functional implication of xenogeneic (human) RelA protein on murine macrophage biology, investigating the transcriptional responses to inflammatory stimuli.

Using confocal imaging, we assessed whether the human RelA subunit can translocate to nucleus upon stimulation with lipopolysaccharide (LPS), implying functional response downstream of Toll-like receptor 4 (TLR4). RNA sequencing analysis was also performed to compare the transcriptional profile of the transgenic BMDM to wild-type C57BL/6J BMDMs, before and post-stimulation using lipid A, the endotoxic centre and immune-activating component of LPS [22]. Validation experiments in BMDMs, at rest and following TLR4 activation, confirmed that the presence of the human *RelA* transgene affects known pro-inflammatory pathways within macrophages and the expression of a variety of genes that are direct or indirect targets of the canonical NFκB pathway. RNA sequencing analysis was also performed on transgenic and C57BL/6J BMDMs stimulated with tumour necrosis factor alpha (TNF), known to be released following LPS-TLR4 activation of macrophages [23]. Comparison of the two stimuli showed that there are ligand-specific differentially affected targets, as well as common genes that are involved in immune responses, such as the interferon and cytokine pathways.

## 2. Materials and Methods

### 2.1. Mice

Transgenic p65-DsRedxp/IκBα-eGFP mice used in this study were generated by the Genomic Technologies Core Facility (University of Manchester, UK) with support of the Max Planck Institute of Molecular Cell Biology and Genetics (Dresden, Germany), as detailed in [10], using previously described NFκB RelA(p65) subunit-optimised *Discosoma* red fluorescent Express protein (DsRedxp) and IκBα-enhanced green fluorescent protein (eGFP) bacterial artificial chromosome (BAC) constructs [24,25]. Mice express fusion proteins of p65-DsRedxp) under the regulation of the native human p65 promoter, and IκBα-eGFP, regulated by the human IκBα promoter, to enable real-time visualisation of human p65 signalling in primary cell cultures and in vivo. Transgenic mice backcrossed on the C57BL/6J background for several generations were made available through the SysmedIBD consortium (www.sysmedibd.eu/ (accessed on 19 February 2022)), with matched C57BL/6J substrain mice provided by Charles River Ltd. (Harlow, UK). 

#### Ethics Statement

Mice were housed at the Biological Services Facility (University of Manchester) under specific-pathogen-free conditions, on a 12 h/12 h light/dark cycle and with access to food and water ad libitum. Transgenic p65-DsRedxp/IκBα-eGFP mice were bred for this study under Home Office project license (PPL 70/7800). Mice were euthanised by rising CO_2_, followed by cervical dislocation, in agreement with the Animal (Scientific Procedures) Act 1986. 

### 2.2. Bone Marrow-Derived Macrophage Isolation and Culture

Bone marrow was isolated from mouse femurs and aliquots prepared in 90% *v*/*v* foetal bovine serum (FBS) containing 10% *v*/*v* dimethyl sulfoxide (DMSO). Fresh isolated bone marrow was differentiated to macrophages for RNA sequencing studies. Vials were also stored in liquid nitrogen, until required for further confocal and qPCR validation studies. Briefly, bone marrow cells (fresh or frozen) were cultured in vitro in the presence of 50 ng/mL recombinant macrophage colony-stimulating factor (MCSF) (PeproTech; London, UK) to induce differentiation of monocytes to macrophages, as described previously [26]. For in vitro stimulation, ligands used included Lipid A (Sigma Aldrich; Poole, UK), LPS extracted using modified phenol/water method [27] from the ileal Crohn’s disease mucosa-associated *Escherichia coli* isolate, LF82 [28], or recombinant mouse tumour necrosis factor alpha (TNF) (Catalogue # 315-01A; PeproTech). 

### 2.3. Confocal Microscopy

BMDMs (2 × 10^5^) from transgenic p65-DsRedxp/IκBα-eGFP mice plated in 35 mm MatTek glass bottom microwells (MatTek Corp.; Ashland, MA, USA), in RPMI 1640 medium containing 10% *v*/*v* FBS. Cultures were stimulated with 100 ng/mL of LPS, and imaged for 5 h using a Leica LSM-800 confocal microscope (488 nm and 561 nm lasers) 

### 2.4. RNA Sequencing 

Unstimulated and Lipid A (100 ng/mL) stimulated BMDM cultures from C57BL/6J and p65-DsRed/IκBα-eGFP mice (*n* = 3) were used for transcriptome analysis. Total RNA was purified using a RNeasy kit (Qiagen). Strand-specific libraries were created with TruSeq stranded Total RNA kits (Illumina; Cambridge, UK) from 1 µg total RNA. RNA sequencing (100-nucleotide paired-end reads) was performed on an Illumina HiSeq2000 platform. 

#### 2.4.1. Read Mapping and Analysis of Differential Expression

Initial data (raw reads) that passed chastity filtering from Illumina sequencing were pre-processed using cutadapt [29] and PrinSeq-lite [30] software and reads aligned to non-repeat masked version of the *Mus musculus* reference genome (GRCm38) using TopHat2 [31], while the corresponding GTF annotation file was obtained from the Ensembl database (*Mus_musculus*.GRCm38.80.gtf). DESeq2 was used for differential expression analysis [32]. Log2 fold changes between C57BL/6J and p65-DsRed/IκBα-eGFP strains were calculated. and adjusted *p*-values corrected for multiple testing (Benjamini and Hochberg method). 

#### 2.4.2. Network and Pathway Analyses

NFκB target genes within the RNA sequencing datasets were identified using an available database from the Gilmore lab (www.bu.edu/nf-kb/gene-resources/target-genes/ (accessed on 4 January 2022)) and key publications [33,34,35,36,37,38,39]. Transcriptional regulatory networks were analysed further using the manually curated reference database TRRUST v2 (www.grnpedia.org/trrust (accessed on 13 January 2022)) [40]. Enrichment analysis of differentially expressed genes was performed using the inBio Discover™ tool (www.inbio-discover.com/ (accessed on 16 January 2022)) to reveal important pathway networks and disease associations [41].

### 2.5. RNA Extraction and qPCR 

Total RNA extracted using the RNeasy mini kit (Qiagen) was reverse transcribed with the High-Capacity RNA-to-cDNA Kit (Applied Biosystems; Paisley, UK). Real time PCR (qPCR) was performed using Taqman Fast advanced master mix and Gene Expression probes, with 50 ng cDNA on a LightCycler 480 qPCR instrument (Roche; Burgess Hill, UK) under standard conditions recommended by the manufacturer (Applied Biosystems). Cp values were calculated from 2nd derivative analysis and relative quantification was calculated using 2^−ΔΔCT^ method [42]. Taqman Gene Expression Assay probes (Applied Biosystems) were *Cxcl10* (Mm00445235_m1), c-*Fos* (Mm00487425_m1), *Il12b* (Mm99999067_m1), *Nfkbia* (Mm00477798_m1), *Ptges* (Mm00452105_m1), *Tnf* (Mm00443258_m1) and *Tnfaip3* (Mm00437121_m1). Results were normalized to *Gapdh* (Mm99999915_g1). 

### 2.6. Statistical Analysis of Experimental Datasets

Statistical inferences on data were performed using Kruskal–Wallis test, followed by all pairwise comparisons of treatments (StatsDirect v3.0.171-StatsDirect Ltd.; Birkenhead, UK). Differences were considered significant when *p* < 0.05. 

## 3. Results

### 3.1. Confocal Imaging of RelA(p65) Translocation in LPS-Stimulated Murine BMDMs

**Figure 1:**
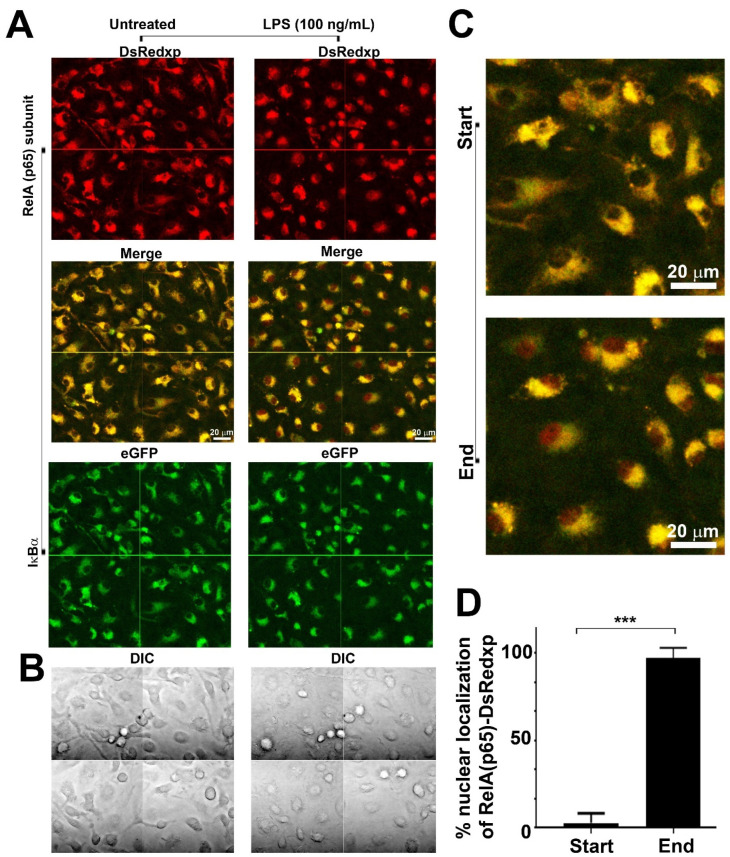
Confocal microscopy shows p65-DsRedxp translocation from the cytoplasm to the nucleus of bone marrow-derived macrophages (BMDMs) from p65-DsRedxp/IκBα-eGFP mice following stimulation with bacterial lipopolysaccharide. Bone marrow cells from frozen stocks were differentiated in vitro to macrophages as described in Materials and Methods. Live macrophages were monitored at rest for 40 min before being stimulated with lipopolysaccharide (LPS), at 100 ng/mL, and continuously monitored for over 5 h. Confocal imaging (using a 63x oil immersion objective) showing composite images of 4 fields, of untreated and post LPS-treatment of BMDMs: (**A**) upper panel, red channel showing p65-DsRedxp localisation; middle panel, red (DsRedxp) and green (eGFP) channels superimposed (Col RG) and, lower panel, green channel, showing IκBα-eGFP localisation in the cytoplasm. (**B**) Differential interference contrast (DIC) imaging of macrophages. (**C**) Higher magnification of the Col RG channel, and (**D**) histogram illustrating % of cells with p65-DsRedxp observed in the nucleus at rest (start), and following TLR4 activation (end); (*n* = 36; *** *p* < 0.001, unpaired *t*-test). Bar = 20 μm.

p65-DsRedxp/IκBα-eGFP BMDMs were cultured in imaging plates and rested overnight in medium containing 10% *v*/*v* FBS. Cells were imaged for 40 min before the addition of the stimulus. At rest, microscopy showed that macrophages expressed (RelA)p65-DsRedxp (red) and IκBα-eGFP (green) within the cytoplasm (Figure 1). Upon stimulation with 100 ng/mL LPS, cells show nuclear translocation of the human RelA signal whilst IκBα-eGFP remains within the cytoplasm (Figure 1). Translocation observed was asynchronous in responding cells and there were also cells that did not seem to respond within the period of observation (see Supplementary Materials, Video S1). 

### 3.2. RNA Sequencing of Bone Marrow-Derived Macrophages Following TLR4 Activation

**Figure 2:**
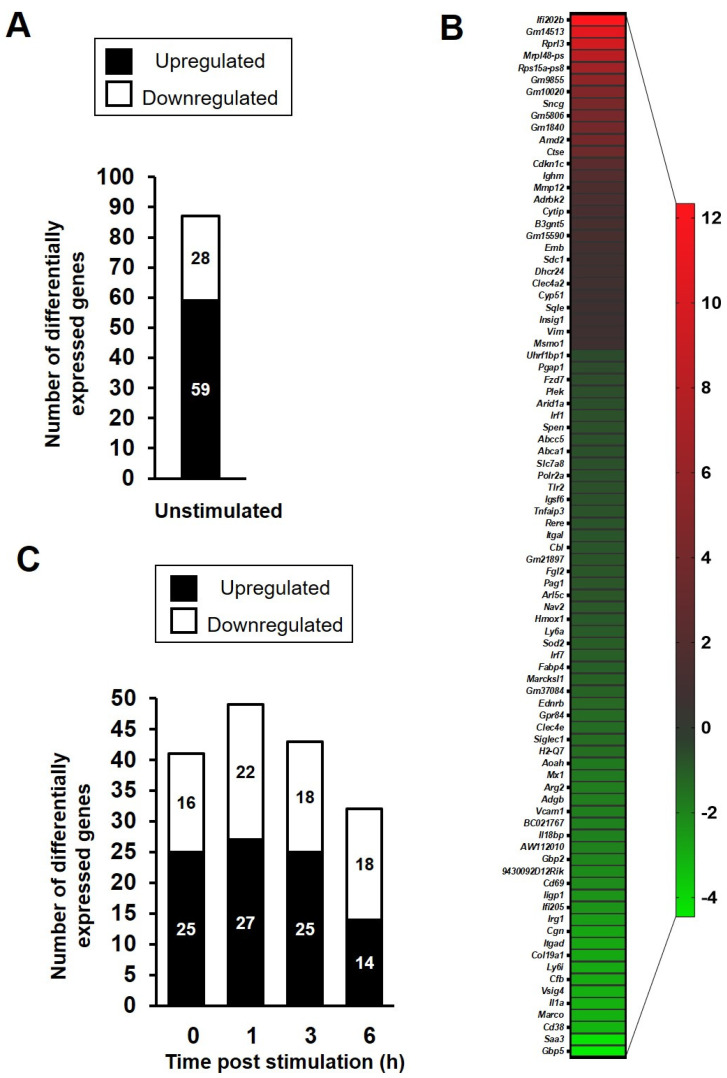
Gene expression changes in p65-DsRedxp/IκBα-eGFP bone marrow-derived macrophages (BMDMs) as identified by RNA sequencing. BMDMs were left unstimulated or stimulated with 100 ng/mL Lipid A for 1, 3, and 6 h (*n* = 3 mice per treatment group). Total RNA was isolated, RNA sequencing was performed, followed by informatics analysis. (**A**) Total number and (**B**) heatmap of 87 differentially expressed (DE) genes (upregulated [red], downregulated [green]) in unstimulated p65-DsRed/IκBα-eGFP BMDMs relative to wild-type C57BL/6J mice, having an adjusted *p*-value of <0.05, corrected for multiple testing using the Benjamini and Hochberg method. (**C**) Number of differentially expressed genes, using a cut-off log2 fold changes ≥1.5 and ≤−1.5, in unstimulated and Lipid A-stimulated p65-DsRedxp/IκBα-eGFP BMDMs (100 ng/mL lipid A, at 1, 3 and 6 h); *n* = 101 genes across all treatment groups.

To further examine the impact of xenogeneic RelA protein in the transcriptional response of murine macrophages to an inflammatory stimulus, both C57BL/6J and p65-DsRedxp/IκBα-eGFP BMDMs (*n* = 3 mice per group) were left untreated or were stimulated with 100 ng/mL Lipid A for a time course of 1, 3 and 6 h. Analysis of the RNA sequencing data revealed that 343 genes were differentially expressed in the transgenic BMDMs at one or more time points relative to wild-type macrophages of the same time point, with an adjusted *p*-value of <0.05. At rest, 87 genes were identified as being differentially expressed in the p65-DsRedxp/IκBα-eGFP BMDMs compared to wild-type macrophages at rest; 28 being upregulated and 59 downregulated (Figure 2A). Median log2 fold change of those genes upregulated at rest was 1.77 (range, 0.644 to 12.34) and those downregulated, −1.32 (range, −4.45 to −0.61); see Figure 2B. 

From the 343 differentially expressed genes, we selected those with a log2 fold change in expression of ≥1.5 and ≤−1.5. When this cut-off filter was applied, 101 genes were identified as having a significant change in expression (Figure 2C). Of these 101 genes, 39 of them were differentially expressed at treatment time points 0 h, 1 h, 3 h and/or 6 h (Supplementary Materials, Figure S1). Further analysis for transcription factor binding, using the manually curated database TRRUST v2, revealed a top five list of transcription factors, in which NFκB1/p105 and RelA/p65 were prominent (Table 1). A search within the differentially expressed gene data set, identified 21 known NFκB target genes (affected in one or more time point), representing ~20% of the total. Out of these genes, 10 genes already showed altered level of expression in unstimulated transgenic BMDMs compared to the wild-type controls; with 9 being reduced in expression (*Saa3* > *Cd38* > *Il1a* > *Cfb* > *Iigp1* > *Cd69* > *Vcam1* > *Mx1* > *Aoah*; range −4.28 to −1.72 log2 fold change) and one increased in expression (*Mmp12*, 1.53 log2 fold change). Notably, amongst these genes identified, *Aoah,* *Cd38* and *Vcam1* encode proteins known to play key roles in macrophage function and/or response to Gram negative bacterial endotoxin [43,44,45]. Upon TLR4 activation, a further 11 NFκB target genes showed differential expression, with *Saa3* being the most decreased in expression in transgenic BMDMs compared to the wild-type controls (−4.12 log2 fold change, at 1 h post-lipid A treatment) and *Ptges* showing the greatest elevation in expression (2.46 log2 fold change, at 6 h post lipid A treatment); see Supplementary Materials, Figure S2).

### 3.3. NFκB Target and Non-Target Genes Are Differentially Expressed in LPS-Stimulated BMDMs

**Figure 3:**
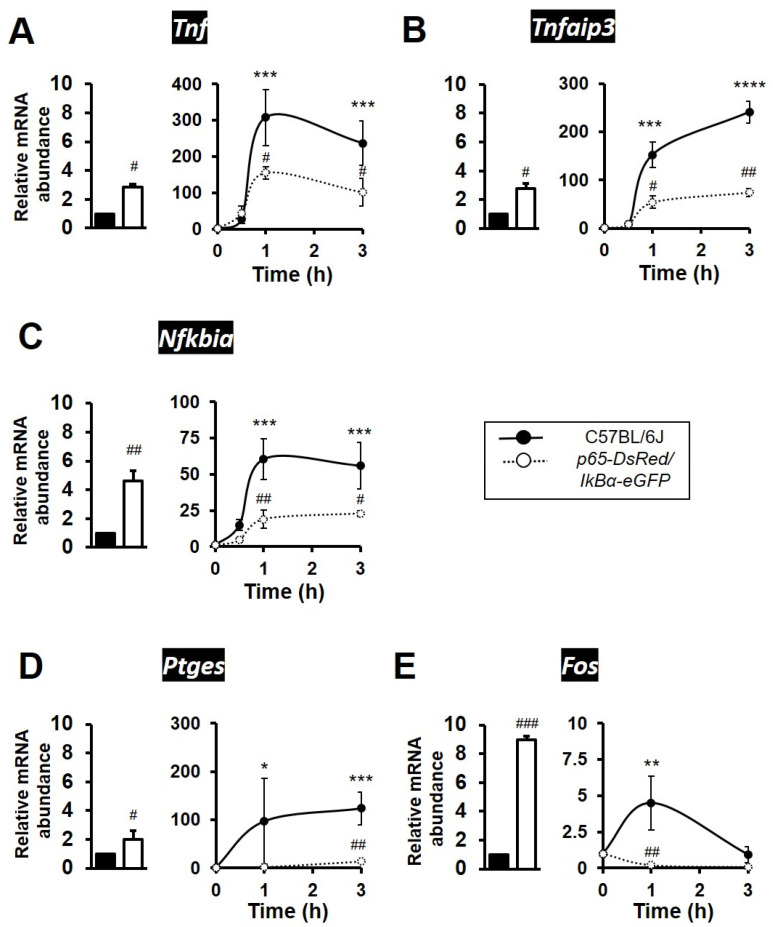
Expression of NFκB target genes are markedly altered in expression in p65-DsRedxp/IκBα-eGFP bone marrow-derived macrophages (BMDMs). Bone marrow cells from frozen stocks were differentiated in vitro to macrophages as described in Materials and Methods. Murine C57BL/6J and transgenic BMDMs were left unstimulated or were stimulated with LPS (100 ng/mL) for a time course of up to 3 h. Total RNA was purified, and the samples were analysed by qPCR for expression of NFκB target genes (**A**) *Tnf*, (**B**) *Tnfaip3*, (**C**) *Nfkbia*, (**D**) *Ptges*, and (**E**) *Fos*; with the left hand panel showing a histogram of fold change in mRNA levels in untreated transgenic BMDMs (white bar) relative to C57BL/6J controls (black bar), and the right hand panel showing dynamic changes in gene expression following treatment with LPS relative to respective unstimulated control or transgenic BMDMs. Significant differences to unstimulated BMDMs, * *p* < 0.05, ** *p* < 0.01, *** *p* < 0.001 and **** *p* < 0.0001; or stimulated transgenic, ^#^ *p* < 0.05, ^##^ *p* < 0.01 and ^###^ *p* < 0.001 (Kruskal–Wallis test; *n* = 3 mice, *n* = 2–3 replicates).

**Figure 4:**
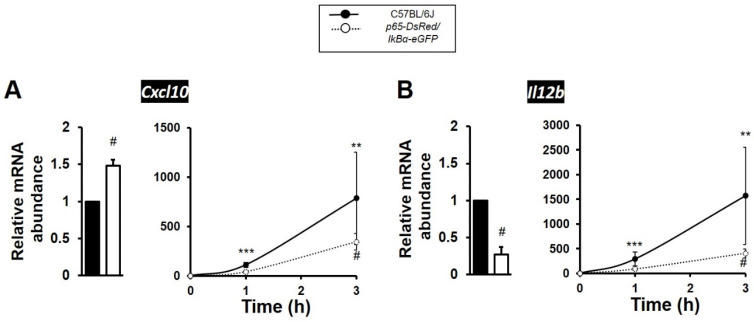
NFκB regulated cytokines/chemokines show decreased gene expression levels in TLR4 activated p65-DsRedxp/IκBα-eGFP bone marrow-derived macrophages (BMDMs). Bone marrow cells from frozen stocks were differentiated in vitro to macrophages as described in Materials and Methods. C57BL/6J and transgenic BMDMs were left unstimulated or were stimulated with LPS (100 ng/mL) for a time course up to 3 h. Total RNA was purified, and the samples were analysed by qPCR for the expression of (**A**) *Cxcl10* and (**B**) *Il12b*; with the left hand panel showing a histogram of fold change in mRNA levels in untreated transgenic BMDMs (white bar) relative to C57BL/6J controls (black bar), and the right hand panel showing dynamic changes in gene expression following treatment with LPS relative to respective unstimulated control or transgenic BMDMs. Significant differences to unstimulated BMDMs, ** *p* < 0.01 and *** *p* < 0.001; or stimulated transgenic, ^#^ *p* < 0.05 (Kruskal–Wallis test; *n* = 3 mice, *n* = 2–3 replicates).

Validation experiments and qPCR analysis showed that presence of the human RelA(p65) subunit in BMDMs resulted in elevated levels of expression under resting conditions for NFκB target genes *Tnf*, *Nfkbia, Tnfaip3* and *Ptges*; all being 2 to 5-fold higher relative to wild-type levels of expression at rest. Similarly, the proto-oncogene *Fos* was ~9-fold higher in expression in unstimulated BMDMs from p65-DsRedxp/IκBα-eGFP mice (Figure 3A–E). Conversely, all 5 genes showed significantly attenuated dynamic levels of expression in the transgenic macrophages stimulated with LPS, as compared to responses seen in TLR4 activated C57BL/6J BMDMs. (Figure 3A–E). Notably, the immediate-early response NFκB target gene *Fos* and the late response gene *Ptges* were markedly reduced, with *Fos* almost undetectable, in LPS-stimulated transgenic strain cultures (Figure 3E). Amongst the late response NFκB target genes, *Cxcl10* (encoding C-X-C motif chemokine ligand 10) showed a statistically significant higher expression at resting state, but its expression was also observed to be 50% lower in the LPS-stimulated p65-DsRedxp/IκBα-eGFP BMDMs compared to stimulated wild-type C57BL/6J macrophages (Figure 4A). *Il12b,* encoding IL-12p40, was also affected, showing 75% reduction in expression levels in the LPS-stimulated p65-DsRedxp/IκBα-eGFP BMDMs at 3 h (Figure 4B). 

### 3.4. Integrative Pathway Enrichment Analysis of Differentially Expressed Genes in p65-DsRed/IκBα-eGFP BMDMs

**Figure 5:**
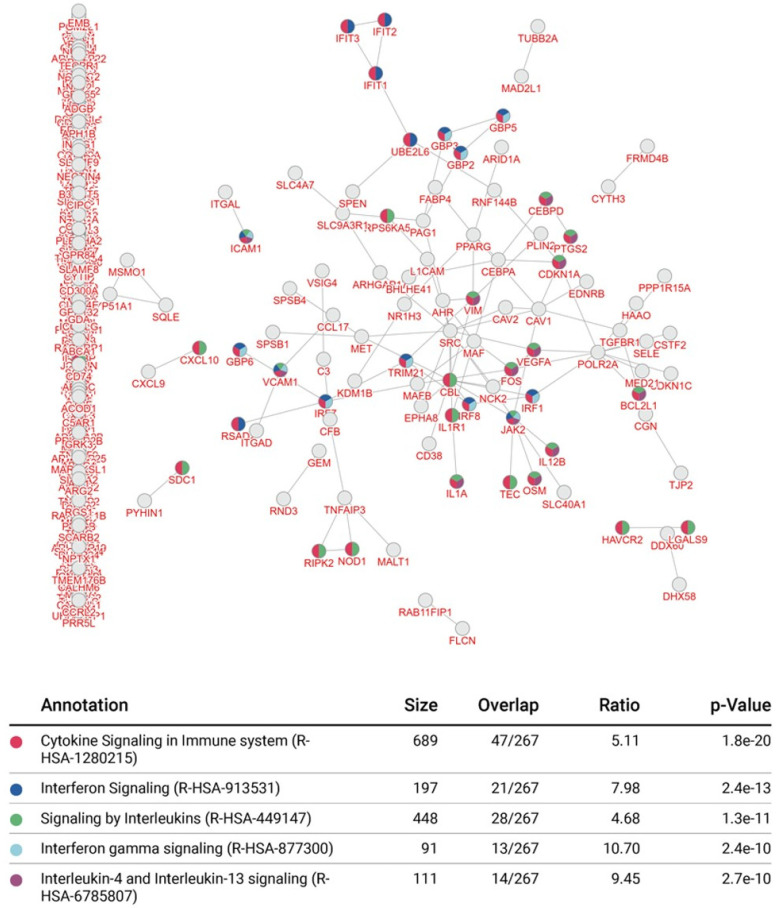
Integrative pathway enrichment analysis in p65-DsRed/IκBα-eGFP BMDMs. The enrichment map and data table indicate the top 5 prognostic signalling pathways identified using all 363 differentially expressed genes identified in unstimulated and Lipid A-stimulated (100 ng/mL, at 1, 3 and 6 h) p65-DsRedxp/IκBα-eGFP BMDMs. Interactions are indicated by connecting lines and multi-coloured nodes indicate pathways that were prognostic according to several types of molecular evidence. Data outputs were generated using the inBio Discover™ tool (www.inbio-discover.com/ (accessed on 16 January 2022)) and no relevance score cut-off was used.

**Figure 6:**
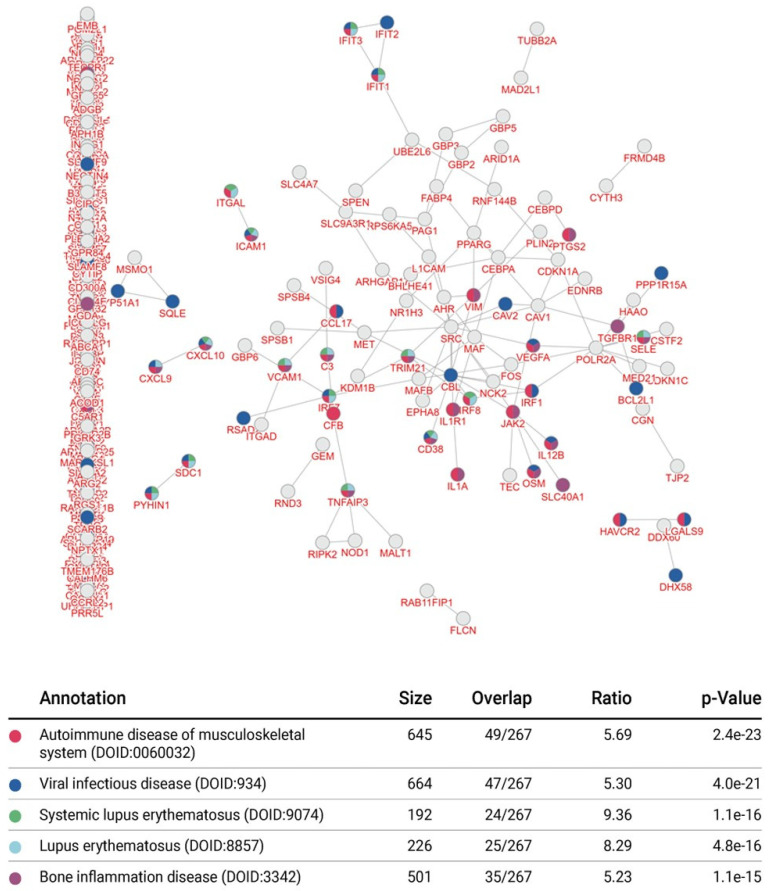
Integrative enrichment disease association analysis as defined by all differentially expressed genes identified in p65-DsRed/IκBα-eGFP BMDMs. The enrichment map and data table indicate the top 5 significant prognostic disease associations from 363 differentially expressed genes identified in unstimulated and Lipid A-stimulated (100 ng/mL, at 1, 3 and 6 h) p65-DsRedxp/IκBα-eGFP BMDMs. Interactions are indicated by connecting lines and multi-coloured nodes indicate prognostic disease processes based on molecular evidence submitted. Data outputs were generated using the inBio Discover™ tool (www.inbio-discover.com/ (accessed on 16 January 2022)) and no relevance score cut-off was used.

Using the inBio Discover™ tool, we performed an enrichment analysis to explore which molecular interactions and relation networks all 343 differentially expressed genes might be involved in. This revealed that amongst the most prominent pathways featured were the interferon and cytokine signalling pathways (Figure 5). Enrichment analysis also revealed that autoimmune disease, inflammatory bone diseases and viral infection were amongst the top 5 conditions linked to the affected proteins in the p65-DsRedxp/IκBα-eGFP macrophages (Figure 6). The analysis was repeated with only those genes with a log2 fold change ≥1.5 and ≤−1.5 (*n* = 101) and a similar outcome was seen (Supplementary Materials, Figure S3). 

### 3.5. Comparison of BMDM Transcriptional Responses to TNFR1 and TLR4 Activation

We further examined the impact of xenogeneic RelA protein on the transcriptional response to TNF in murine C57BL/6J and p65-DsRedxp/IκBα-eGFP BMDMs (30 ng/mL TNF for 1, 3 and 6 h; *n* = 3 mice per group). Analysis of the RNA sequencing data following TNFR1 activation revealed that 550 genes were differentially expressed in the transgenic BMDMs at one or more time points, relative to wild-type macrophages (at 1 h TNF, *n* = 210; at 3 h TNF, *n* = 173 and at 6 h TNF, 167 genes). Of these, 222 genes were identified as having a significant change in expression with a log2 fold change ≥ ±1.5, with 146 of them were differentially expressed at one or more TNF treatment time points (1 h, 3 h and/or 6 h) and 36 known NFκB target genes, representing ~25% of the total (Supplementary Materials, Figure S4).

**Figure 7:**
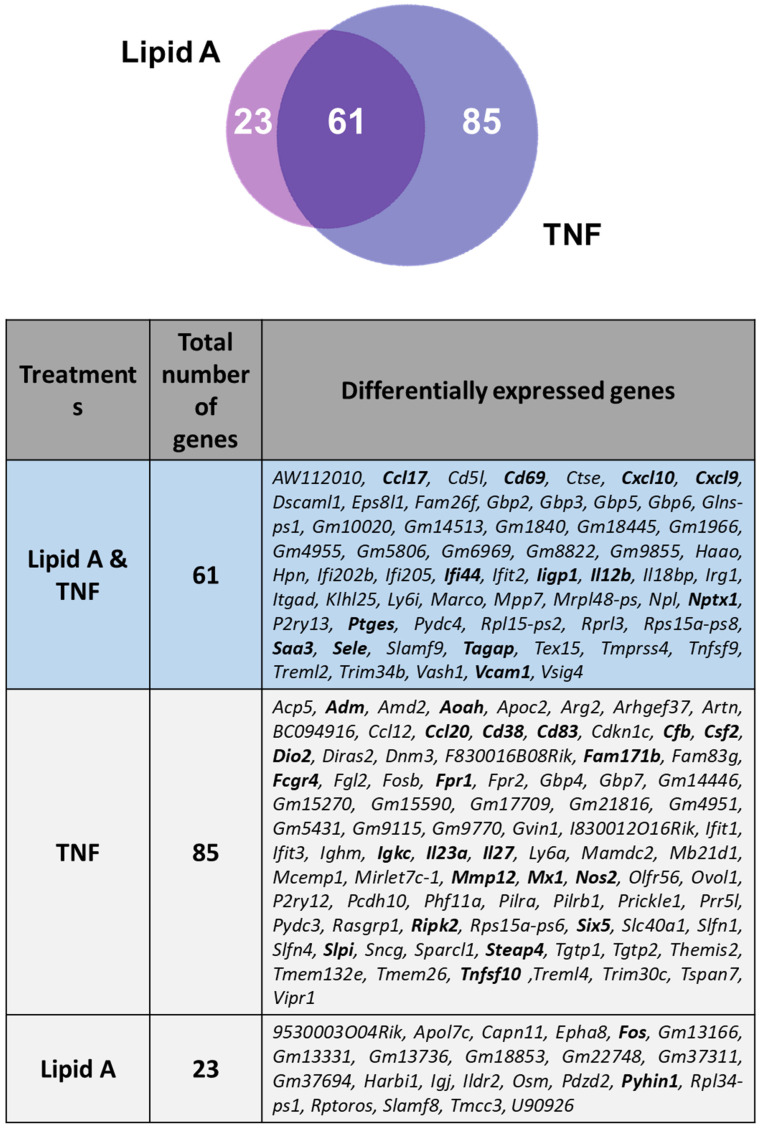
Distribution of differentially expressed genes comparing Lipid A and TNF treatments of p65-DsRedxp/IκBα-eGFP BMDMs. Venn diagram illustrating the distribution of differentially expressed genes common to Lipid A-stimulated and TNF-stimulated BMDMs (100 ng/mL Lipid A or TNF 30 ng/mL for 1, 3, and 6 h; *n* = 3 mice per treatment group). Total RNA was isolated, RNA sequencing was performed, followed by informatics analysis. All genes included were identified as being significantly changed in expression (*p* < 0.05, corrected for multiple testing using the Benjamini and Hochberg method, and based on log2 fold changes ≥1.5 and ≤−1.5) compared to treated wild-type control mice. Weighted Venn diagram constructed using Biovenn (www.biovenn.nl (accessed on 14 March 2022)) [46] and table using Venn diagram software from Ghent University, freely available at http://bioinformatics.psb.ugent.be/webtools/Venn/ (accessed on 28 January 2022). NFκB target genes identified from database searches are indicated in bold.

Comparison between Lipid A-treated and TNF-treated p65-DsRedxp/IκBα-eGFP BMDMs showed a significant proportion of overlapping genes, ~36% overall, with 13/61 being NFκB target genes (Figure 7). Enrichment pathway analysis of the 61 overlapping differentially expressed genes was conducted using inBio Discover tool, revealing common affected immune response pathways (Supplementary Materials, Figure S5).

## 4. Discussion

The NFκB family of transcription factors consists of proteins that are ubiquitously expressed and are involved in a wide range of biological functions. In macrophages, NFκB activation can be induced downstream a variety of pattern recognition receptors (PRRs), such as TLR4, and cytokine receptors, such as TNFR1, both regulating the function of macrophages in innate and adaptive immune responses [47]. Here, we studied TLR4 pathway activation in p65-DsRedxp/IκBα-eGFP murine macrophages expressing the human RelA(p65) subunit. Human RelA showed cytoplasmic distribution in resting macrophages and LPS-induced nuclear translocation within the first 120 min of activation. Our observations here are in agreement with previous reports that describe a single translocation wave in macrophages downstream of TLR4 activation [47].

One of the first studies to use a transgenics approach with specific aim to evaluate NFκB activity, showed that the NFκB/Rel family of transcriptional activators were involved in tissue-specific and inducible gene activation [48]. A few years later, a lacZ reporter mouse driven by promoter elements that were dependent on the presence of nuclear NFκB/Rel activity, indicated that NFκB was unlikely to be involved in regulating processes of early development and differentiation of the different tissues, but rather it had greater importance in maintaining their function once cells had matured [49]. More recently, studies using transgenic mice to study thymic development has confirmed that the classical NFκB pathway is indeed responsible for development of specific T cell populations [50]. Also, a p65 S276A knock-in mouse, in p65 cannot be phosphorylated on serine 276, not only caused embryonic lethality, but it also affected expression of genes not normally regulated by NFκB [51]. Therefore, it is of key importance to evaluate any new transgenic NFκB strain in depth before proceeding to complex in vivo experimentation. The p65-DsRedxp reporter mouse has previously been used for live fluorescence imaging of mouse intervertebral disc (IVD) explants, where it was shown that the RelA(p65) subunit translocated to nucleus of cells within the IVD following stimulation with IL-1β or TNF [20]. In our own earlier studies using the p65-DsRedxp/IκBα-eGFP double transgenic strain, we successfully showed that TNF induced p65 oscillations in murine intestinal-stem cell derived 3D organoid cultures [21]. In those experiments, we observed a first, synchronised wave of p65 nuclear translocation, followed by a second wave of partially synchronised translocation [21]. In the current study, we observed one wave of nuclear translocation in p65-DsRedxp/IκBα-eGFP macrophages upon LPS stimulation. Moreover, in support of this, we have previously shown that upon LPS treatment only a single, strong nuclear translocation of p65 is observed by confocal imaging of human blood-derived macrophages expressing human p65-AmCyan [52], and in lipid A-induced murine p65-DsRedxp BMDMs [10]. Again, these observations in human and murine macrophages are in agreement with cell-specific observations of p65 activation profiles [47]. 

Given, in our experiments, that the human p65 protein showed inducible nuclear translocation, we set up key experiments to explore its influence on the transcriptional profile of transgenic BMDMs, under resting conditions and upon TLR4 stimulation. Over 300 genes were differentially regulated in the p65-DsRedxp/IκBα-eGFP cells with 20% of them identified as known NFκB targets. Validation experiments confirmed the impact of the human p65 protein on some of these differentially expressed genes, where they showed similar expression profiles, but markedly reduced mRNA abundance levels compared to the wild-type cells. This could be due to primary sequence specific differences in RelA between the two species. Comparing the human and mouse RelA protein sequences reveals differences in the carboxy terminal domain, as previously reported [53]. It is of note too that the linker region between the Rel Homology Domain (RHD) and the Transactivation Domain (TAD) shows low homology between the human and mouse sequences. There is also a small stretch of non-conserved amino acids (IPVAPH) between conserved region 2 (CR2) and CR3 (aa 473–480 in the human sequence) [53]. The carboxyterminal region of RelA is a transactivation domain regulating protein-protein interactions with transcriptional machinery and other key transcription factors. This could potentially impact the human p65 protein within murine cells. We think it unlikely that the differences can be attributed to presence of the human promotor. Activation dynamics of p65 controlled by the human RelA promotor in BMDMs [10], has been shown to be comparable to those activation profiles of murine p65-EGFP BMDMs where the endogenous p65 locus was tagged [54]. It is possible, however, that overexpression of human IκBα in this transgenic mouse could impact on gene transcription. Previous in vitro studies have looked at the effect of IκBα levels on p65 nuclear translocation [55]. Using co-transfected versus single transfected cells, it was shown that cells expressing markedly higher levels of IκBα-EGFP demonstrated not only slower rates of IκBα-EGFP degradation but also significantly delayed p65-DsRed translocation compared to cells expressing lower levels [55]. Whilst the p65/IκBα ratio can be easily controlled in transient transfection experiments, it is far more difficult to regulate this ratio in an in vivo context, taking in to account that these mice are already heterozygous for each transgene.

Amongst those NFκB target genes observed to be significantly attenuated in expression in LPS-stimulated transgenic macrophages, we identified *Tnf* and *Il12b*, encoding proinflammatory cytokines TNF and IL-12p40. The latter is one of the subunits of bioactive proinflammatory cytokines IL-12p70 and IL-23 that regulate Th1 responses during infection and inflammation [56]. We also confirmed reduced expression of the chemokine Cxcl10 (also known as interferon-gamma (IFN-γ) inducible protein 10), a protein that binds to its receptor CXCR3 and regulates immune responses (mainly Th1) through recruitment of leukocytes, including T cells and monocytes/macrophages [57]. In contrast, a previous study showed expression levels of *Cxcl10* to be normal in LPS-stimulated BMDMs expressing a GFP-p65 fusion protein from the endogenous p65 genomic locus, i.e., where there is no xenogeneic p65 subunit present [54]. In our study, we also observed that the level of expression of *Cd38*, encoding for the cell-surface membrane receptor CD38, was also reduced in p65-DsRedxp/IκBα-eGFP macrophages at rest. CD38 is known to be involved in proinflammatory responses regulating the secretion of inflammatory cytokines, such as IL-12p40, thus impacting on macrophage function [58]. CD38 also has nicotinamide adenine dinucleotide nucleosidase (NADase) activity, and is reported to play key senescence associated function in non-activated macrophages [43]. Vascular cell adhesion protein 1 gene *Vcam1* was also reduced throughout our lipid A stimulation time course. A decrease in Vcam1 levels could have a substantial impact in vivo because it has been shown to regulate trans-endothelial migration of macrophages during inflammation [59], and more recently, was shown to be involved in homing of haematopoietic stem and progenitor cells [44]. Another gene identified as decreased in expression in resting macrophages was *Aoah*, encoding for the enzyme acyloxyacyl hydrolase, an important lipase that inactivates Gram-negative bacterial endotoxin (LPS) [45]. Additionally, we noted reduced relative mRNA abundance levels of *Nfkbia* and *Tnfaip3*, encoding for IκBα and A20 respectively, both downstream targets and known inhibitors of the NFκB pathway [60,61,62]. Comparison of all differentially regulated genes in the DsRedxp/IκBα-eGFP transgenic macrophages downstream of TLR4 and TNFR1 activation showed common genes affected. This is not surprising, given that it has already been shown that the two receptors have common downstream signalling pathways in macrophages [63]. Pathway analysis undertaken based on genes differentially expressed in both LPS and TNF treated p65-DsRedxp/IκBα-eGFP macrophages revealed their importance in cytokine and IFN-γ signalling pathways. 

## 5. Conclusions

Taken together, these data suggest that the presence of the xenogeneic RelA(p65) protein in murine p65-DsRedxp/IκBα-eGFP macrophages likely has an inhibitory action. We speculate that this might either be due to competition with endogenous p65 for (i) dimerization with other NFκB subunits and/or (ii) interaction with other key transcriptional regulators. Similarly, it is also possible that overexpression the human IκBα could impact on the signalling pathway that regulates activation of NFκB dimers, and therefore downstream gene transcription. Given that the p65-DsRedxp/IκBα-eGFP macrophages have reduced specific transcriptional profiles of key molecules involved in innate and adaptive immunity, they are highly likely to show defective Th1 responses. It is therefore essential that this information be taken into consideration when designing future experiments and/or interpreting phenotypes during experimental protocols. 

## Figures and Tables

**Table 1:** Transcriptional regulatory relationships identified from the differentially expressed genes in bone marrow-derived macrophages (BMDMs) from p65-DsRedxp/IκBα-eGFP transgenic mice.

TRRUST ^1^ v.2 Database Analysis				
Transcription Factor	Description	Number of Genes	*p*-Value	FDR ^2^
Nfkb1	Nuclear factor of kappa light polypeptide gene enhancer in B cells 1, p105	9	7.9 × 10^−9^	8.74 × 10^−8^
Irf1	Interferon regulatory factor 1	4	8.8 × 10^−7^	4.85 × 10^−6^
Stat1	Signal transducer and activator of transcription 1	4	3.2 × 10^−6^	1.16 × 10^−5^
RelA(p65)	v-rel reticuloendotheliosis viral oncogene homolog A (avian)	5	3.1 × 10^−5^	8.58 × 10^−5^
Mafb	v-maf musculoaponeurotic fibrosarcoma oncogene family, protein B (avian)	2	8.8 × 10^−5^	1.61 × 10^−4^

^1^ TRRUST, Transcriptional Regulatory Relationships Unravelled by Sentence-based Text mining; ^2^ FDR, False discovery rate.

## Data Availability

All data generated or analysed during this study are included in this published article and the Supplementary Materials. RNA sequencing data has been deposited in the ArrayExpress database at EMBL-EBI (www.ebi.ac.uk/arrayexpress (accessed on 12 February 2022)) under accession number E-MTAB-11461.

## Supplementary Materials

The following supporting information can be downloaded at: https://www.mdpi.com/article/10.3390/biomedicines10040757/s1, Video S1: RelA(p65) translocation from the cytoplasm to the nucleus of bone marrow-derived macrophages (BMDMs) from p65-DsRedxp/IκBα-eGFP mice following stimulation with bacterial lipopolysaccharide. Figure S1: Distribution of differentially expressed genes in p65-DsRedxp/IκBα-eGFP BMDM treatment groups, as identified by RNA sequencing. Figure S2: Differentially expressed genes identified by RNA sequencing in p65-DsRedxp/IκBα-eGFP bone marrow derived murine macrophages (BMDMs), with or with treatment with Lipid A. Figure S3: Integrative pathway enrichment analysis of differentially expressed genes from p65-DsRed/IκBα-eGFP BMDMs using a cut-off of ≥1.5 and ≤−1.5 log2 fold change in expression. Figure S4: Number and distribution of differentially expressed genes identified by RNA sequencing in p65-DsRedxp/IκBα-eGFP bone marrow derived murine macrophages (BMDMs), with or with treatment with tumour necrosis factor. Figure S5: Integrative pathway enrichment analysis of differentially expressed genes common to Lipid A and TNF treatment of p65-DsRedxp/IκBα-eGFP BMDMs.
