# Supplementary material for: Effects of Human RelA Transgene on Murine Macrophage Inflammatory Responses
Source: Biomedicines. 2022 Mar 24;10(4):757. doi: 10.3390/biomedicines10040757 (PMC9027775; doi:10.3390/biomedicines10040757)
Supplement: Supplementary file 1 [file biomedicines-10-00757-s001.zip › Supplementary Materials - Figure S1.pdf]

Effects of human RelA transgene on murine macrophage inflammatory responses. Papoutsopoulou S, Morris L, Bayliff A, Mair T, Stagi M, Bergey F, Alam MT, Sheibani-Tezerji R, Rosenstiel P, Müller W, Martins Dos Santos VAP, Campbell BJ.

SUPPLEMENTARY MATERIALS

Figure S1: Distribution of differentially expressed genes identified in p65-DsRedxp/IκBα-eGFP BMDMs by RNA sequencing.

Venn diagram illustrating the distribution of differentially expressed genes amongst untreated and Lipid A-stimulated BMDMs (100 ng/mL Lipid A for 1, 3, and 6 h; N = 3 mice per treatment group). Total RNA was isolated, RNA sequencing was performed, followed by informatics analysis. Differentially expressed genes were identified as being significantly changed in expression (log2 fold change ≥ 1.5 and ≤ -1.5, and p<0.05; corrected for multiple testing using the Benjamini and Hochberg method) between p65-DsRedxp/IκBα-eGFP macrophages and WT controls at each treatment timepoint. Venn diagram software from Ghent University, freely available at http://bioinformatics.psb.ugent.be/webtools/Venn/.

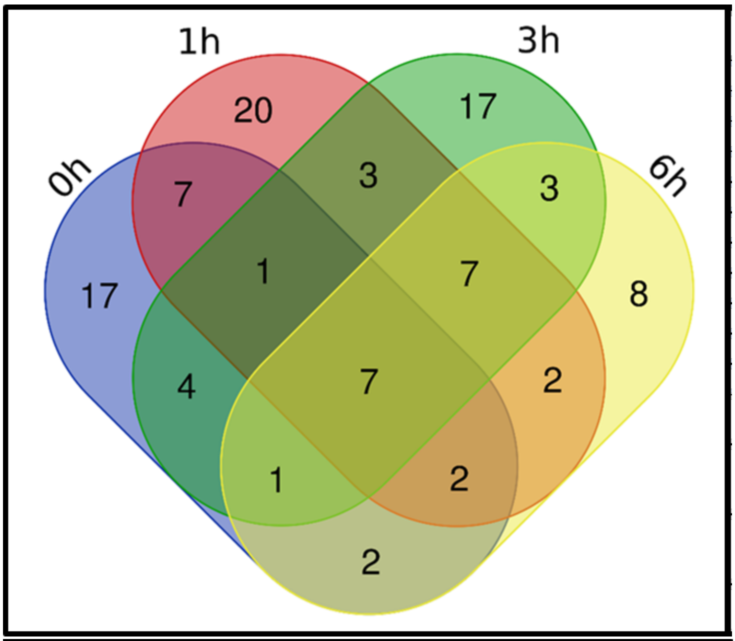

Treatment groups	Total number of genes	Differentially expressed genes
0h 1h 3h 6h	7	*Gm1840, Ctse, Gm5806, Rprl3, Gm14513, Gbp5, Ifi202b*
0h 1h 3h	1	*Marco*
0h 1h 6h	2	*Cd69, Vcam1*
0h 3h 6h	1	*Gm9855*
1h 3h 6h	7	*Npl, Gm8822, Gm18445, Gm6969, Nptx1, Rpl15-ps2, Cxcl9*
0h 1h	7	*AW112010, Gbp2, Saa3, Irg1, Il18bp, Iigp1, Ifi205*
0h 3h	4	*Itgad, Gm10020, Vsig4, Ly6i*
0h 6h	2	*Rps15a-ps8, Mrpl48-ps*
1h 3h	3	*Ifi44, Glns-ps1, Gbp6*
1h 6h	2	*Haao, Eps8l1*
3h 6h	3	*Ptges, Fam26f, P2ry13*
0h	17	*Cd38, Amd2, Cdkn1c, Il1a, Aoah, Cfb, Ighm, Cgn, Mmp12, Sncg, 9430092D12Rik, Mx1, Adrbk2, Adgb, Arg2, Col19a1, BC021767*
1h	20	*Gbp3, Rptoros, Osm, Hpn, Fos, Ifit2, Pydc4, Cxcl10, Gm13736, Rpl34-ps1, Ildr2, Gm13331, Tagap, Gm4955, Gm22748, Igj, Slamf8, Epha8, Pyhin1, Il12b*
3h	17	*Mpp7, Tmprss4, Tmcc3, 9530003O04Rik, Tex15, Tnfsf9, Gm37694, Apol7c, Treml2, Harbi1, Gm37311, Sele, Capn11, Dscaml1, Ccl17, Pdzd2, Vash1*
6h	8	*Cd5l, Trim34b, Slamf9, Klhl25, Gm18853, Gm1966, U90926, Gm13166*
