# Supplementary material for: Effects of Human RelA Transgene on Murine Macrophage Inflammatory Responses
Source: Biomedicines. 2022 Mar 24;10(4):757. doi: 10.3390/biomedicines10040757 (PMC9027775; doi:10.3390/biomedicines10040757)
Supplement: Supplementary file 1 [file biomedicines-10-00757-s001.zip › Supplementary Materials - Figure S2.pdf]

Effects of human RelA transgene on murine macrophage inflammatory responses. Papoutsopoulou S, Morris L, Bayliff A, Mair T, Stagi M, Bergey F, Alam MT, Sheibani-Tezerji R, Rosenstiel P, Müller W, Martins Dos Santos VAP, Campbell BJ.

SUPPLEMENTARY MATERIALS

Figure S2: Differentially expresssed genes identified by RNA sequencing in p65-DsRedxp/IκBα-eGFP bone marrow derived murine macrophages (BMDMs). Significant differentially expressed genes identified by RNAseq using a cut-off ≥ 1.5 and ≤ -1.5 log2 fold change (FC) in expression, between unstimulated controls and Lipid A-stimulated BMDMs (100 ng/mL, at 1, 3 and 6 h); n=101 genes across all treatment groups. Heatmap of **(A)** up-regulated genes (red), and **(B)** downregulated genes (green), in untreated and TNF-treated p65-DsRedxp/IκBβ-eGFP BMDMs relative to wild-type C57BL/6J macrophages. Genes highlighted in black boxes were identified as NFκB target genes based on database searches.

A

Untreated		1 h post Lipid A		3h post Lipid A		6h post Lipid A	
**Gene**	**Log$_2$ FC**	**Gene**	**Log$_2$ FC**	**Gene**	**Log$_2$ FC**	**Gene**	**Log$_2$ FC**
Ifi202b	12.34	*Ifi202b*	11.16	*Rprl3*	9.73	*Ifi202b*	11.67
Gm14513	10.64	*Gm14513*	10.59	*Ifi202b*	9.06	*Gm14513*	10.21
Rprl3	9.56	*Rprl3*	10.09	*Gm14513*	7.79	*Rprl3*	9.61
Mrpl48-ps	8.16	*Glns-ps1*	5.84	*Gm10020*	6.93	*Gm5806*	7.43
Rps15a-ps8	6.79	*Rpl15-ps2*	5.80	*Rpl15-ps2*	5.91	*Rps15a-ps8*	6.69
Gm9855	5.62	*Igj*	5.45	*Gm9855*	5.64	*Rpl15-ps2*	5.89
Gm10020	4.75	*Gm1840*	5.21	*Ctse*	4.99	*Gm1840*	5.02
Sncg	4.22	*Ctse*	4.52	*Glns-ps1*	4.33	*Gm9855*	4.59
Gm5806	4.21	*Epha8*	4.50	*Gm1840*	4.19	*Ctse*	4.25
Gm1840	4.02	*Eps8l1*	4.44	*Gm5806*	3.87	*Trim34b*	4.23
Amd2	3.96	*Gm18445*	4.19	*Gm8822*	3.33	*Mrpl48-ps*	3.84
Ctse	3.56	*Gm13736*	3.98	*Capn11*	2.89	*Eps8l1*	3.74
Cdkn1c	2.43	*Gm5806*	3.93	*Gm18445*	2.82	*Gm18445*	3.23
Ighm	2.00	*Gm13331*	3.53	*Mpp7*	1.98	*Gm8822*	2.46
Mmp12	1.53	*Rpl34-ps1*	3.38	*Npl*	1.86	***Ptges***	2.46
Adrbk2	1.50	*Ildr2*	3.06	***Ptges***	1.80	*U90926*	2.13
		Gm8822	2.57	*Harbi1*	1.61	*Npl*	2.08
		Gm22748	2.10	***Nptx1***	1.52	***Nptx1***	2.01
		Nptx1	2.01				
		Npl	1.98				
		Osm	1.72				
		Fos	1.53				

B

Untreated		1 h post Lipid A		3h post Lipid A		6h post Lipid A	
Gene	Log_2 FC	Gene	Log_2 FC	Gene	Log_2 FC	Gene	Log_2 FC
**Aoah**	-1.72	**Tagap**	-1.50	Gm37311	-1.52	Klhl25	-1.51
**Mx1**	-1.75	Slamf8	-1.52	Apol7c	-1.54	**Vcam1**	-1.52
Arg2	-1.82	**Pyhin1**	-1.53	**Ifi44**	-1.56	**Cd69**	-1.52
Adgb	-1.85	Ifit2	-1.60	P2ry13	-1.59	Fam26f	-1.65
**Vcam1**	-1.88	**Ifi44**	-1.61	Vash1	-1.59	Cd5l	-1.88
BC021767	-1.93	Irg1	-1.64	Gbp5	-1.59	Gbp5	-1.89
Il18bp	-1.94	Hpn	-1.72	Tmcc3	-1.62	Gm1966	-1.93
AW112010	-1.94	**Cd69**	-1.73	Marco	-1.64	Slamf9	-2.24
Gbp2	-2.05	Il18bp	-1.78	Fam26f	-1.75	**Cxcl9**	-2.28
9430092D12Rik	-2.17	**Cxcl10**	-1.83	**Sele**	-1.78	Gm18853	-2.44
**Cd69**	-2.19	Pydc4	-1.95	Tnfsf9	-1.78	P2ry13	-2.98
**Iigp1**	-2.39	Gbp3	-1.97	Tex15	-1.80	Gm13166	-3.48
Ifi205	-2.39	**Iigp1**	-1.99	Gbp6	-1.87	Gm6969	-4.88
Irg1	-2.57	AW112010	-2.06	Treml2	-1.88	Haao	-4.96
Cgn	-2.82	Gm4955	-2.22	9530003O04Rik	-1.92		
Itgad	-2.84	**Vcam1**	-2.39	**Cxcl9**	-1.99		
Col19a1	-2.86	Gbp2	-2.44	Gm37694	-2.16		
Ly6i	-2.93	Gbp6	-2.51	Ly6i	-2.24		
**Cfb**	-2.97	Ifi205	-2.85	**Ccl17**	-2.53		
Vsig4	-3.04	Gm6969	-3.11	Tmprss4	-2.66		
**Il1a**	-3.08	Marco	-3.15	Pdzd2	-2.68		
Marco	-3.12	Rptoros	-3.19	Gm6969	-3.00		
**Cd38**	-3.19	**Il12b**	-3.38	Itgad	-3.36		
**Saa3**	-4.28	**Cxcl9**	-3.39	Dscaml1	-3.59		
Gbp5	-4.45	Haao	-3.50	Vsig4	-4.09		
		**Saa3**	-4.12				
		Gbp5	-4.20				
