# Supplementary material for: Effects of Human RelA Transgene on Murine Macrophage Inflammatory Responses
Source: Biomedicines. 2022 Mar 24;10(4):757. doi: 10.3390/biomedicines10040757 (PMC9027775; doi:10.3390/biomedicines10040757)
Supplement: Supplementary file 1 [file biomedicines-10-00757-s001.zip › Supplementary Materials - Figure S3.pdf]

Effects of human RelA transgene on murine macrophage inflammatory responses. Papoutsopoulou S, Morris L, Bayliff A, Mair T, Stagi M, Bergey F, Alam MT, Sheibani-Tezerji R, Rosenstiel P, Müller W, Martins Dos Santos VAP, Campbell BJ.

SUPPLEMENTARY MATERIALS

Figure S3: Integrative pathway enrichment analysis of differentially expressed genes from p65-DsRedxp/IκBα-eGFP BMDMs using a cut-off ≥ 1.5 and ≤ -1.5 log2 fold change in expression. Enrichment maps and data tables indicate the top five prognostic **(A)** signalling pathways and **(B)** disease associations identified in unstimulated and Lipid A-stimulated p65-DsRed/IκBα-eGFP BMDMs (100 ng/mL, at 1, 3 and 6 h); n=101 genes across all treatment groups. Interactions are indicated by connecting lines and multi-coloured nodes indicate pathways or disease associations that were prognostic based on molecular evidence submitted. Data outputs were generated using the inBio Discover™ tool (www.inbio-discover.com/) and no relevance score cut-off was used.

A

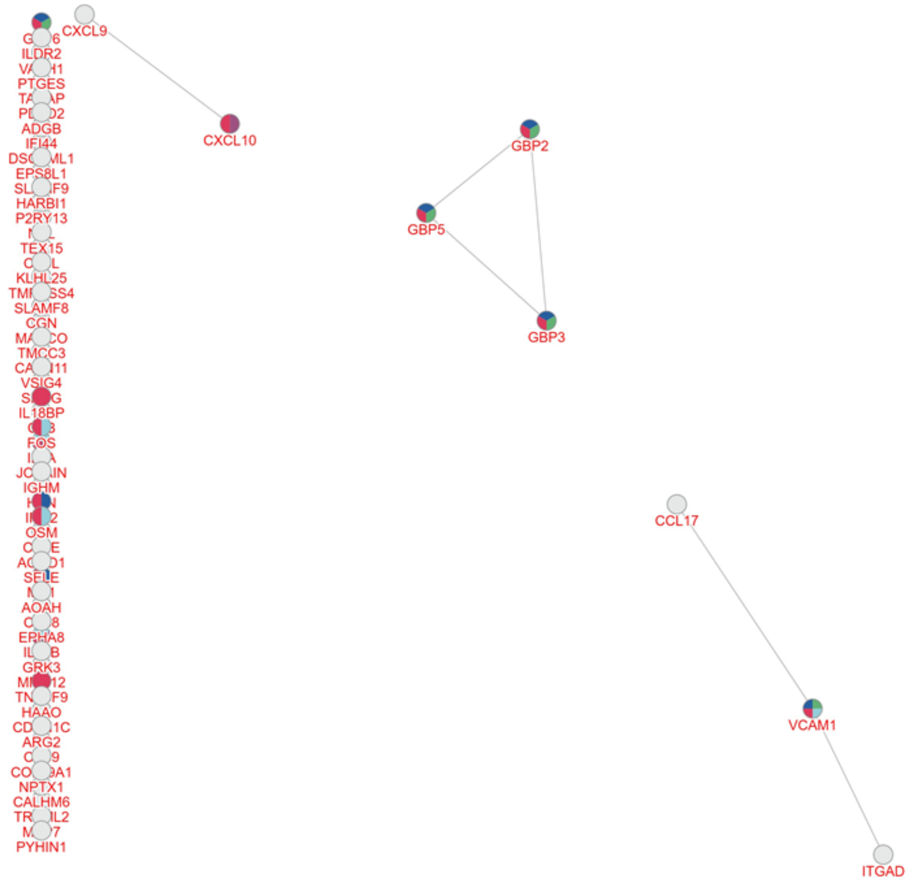

Annotation	Size	Overlap	Ratio	p-Value
Cytokine Signaling in Immune system (R-HSA-1280215)	689	14/63	6.45	2.3e-8
Interferon Signaling (R-HSA-913531)	197	7/63	11.28	2.8e-6
Interferon gamma signaling (R-HSA-877300)	91	5/63	17.44	1.0e-5
Interleukin-4 and Interleukin-13 signaling (R-HSA-6785807)	111	5/63	14.30	2.6e-5
Interleukin-10 signaling (R-HSA-6783783)	45	3/63	21.16	3.8e-4

B

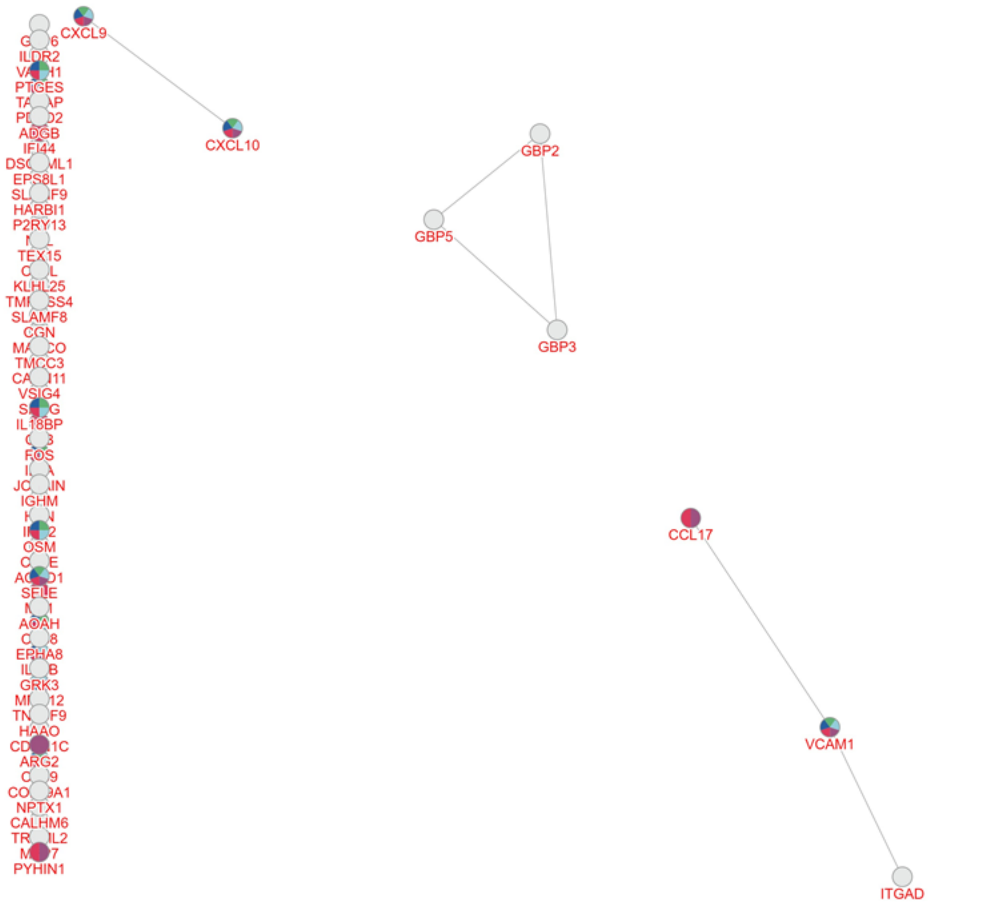

Annotation	Size	Overlap	Ratio	p-Value
Autoimmune disease of musculoskeletal system (DOID:0060032)	645	17/63	8.37	9.1e-12
Arthritis (DOID:848)	481	13/63	8.58	2.7e-9
Rheumatoid arthritis (DOID:7148)	313	11/63	11.16	3.5e-9
Bone inflammation disease (DOID:3342)	501	13/63	8.24	4.4e-9
Bronchial disease (DOID:1176)	252	10/63	12.60	6.0e-9
