# Supplementary material for: Effects of Human RelA Transgene on Murine Macrophage Inflammatory Responses
Source: Biomedicines. 2022 Mar 24;10(4):757. doi: 10.3390/biomedicines10040757 (PMC9027775; doi:10.3390/biomedicines10040757)
Supplement: Supplementary file 1 [file biomedicines-10-00757-s001.zip › Supplementary Materials - Figure S4.pdf]

Effects of human RelA transgene on murine macrophage inflammatory responses. Papoutsopoulou S, Morris L, Bayliff A, Mair T, Stagi M, Bergey F, Alam MT, Sheibani-Tezerji R, Rosenstiel P, Müller W, Martins Dos Santos VAP, Campbell BJ.

SUPPLEMENTARY MATERIALS

Figure S4: Number and distribution of differentially expressed genes identified by RNA sequencing in p65-DsRedxp/IκBα-eGFP bone marrow-derived murine macrophages (BMDMs), with or with treatment with tumour necrosis factor (TNF).

Total RNA was isolated from untreated and TNF-stimulated BMDMs (30 ng/mL TNF for 1, 3, and 6 h; N = 3 mice per treatment group). RNA sequencing was performed, followed by informatics analysis. **(A)** Total number of differentially expressed genes identified as being significantly upregulated and downregulated following treatment in p65-DsRed/IκBα-eGFP BMDMs relative to wild-type C57BL/6J mice (using a cut-off ≥ 1.5 and ≤ -1.5 log2 fold change in expression, and *p*<0.05; corrected for multiple testing using the Benjamini and Hochberg method. **(B)** Venn diagram showing distribution of DE genes, generated using freely available software from Ghent University, at http://bioinformatics.psb.ugent.be/webtools/Venn/. Heatmap of **(C)** up-regulated genes (red), and **(D)** downregulated genes (green), in untreated and TNF-treated p65-DsRedxp/IκBα-eGFP BMDMs relative to wild-type C57BL/6J macrophages. Genes highlighted in black boxes were identified as NFκB target genes based on database searches.

A

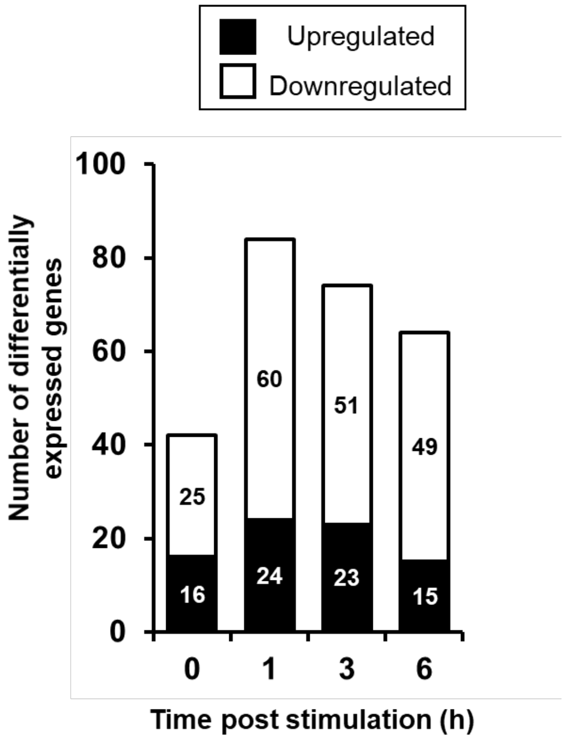

B

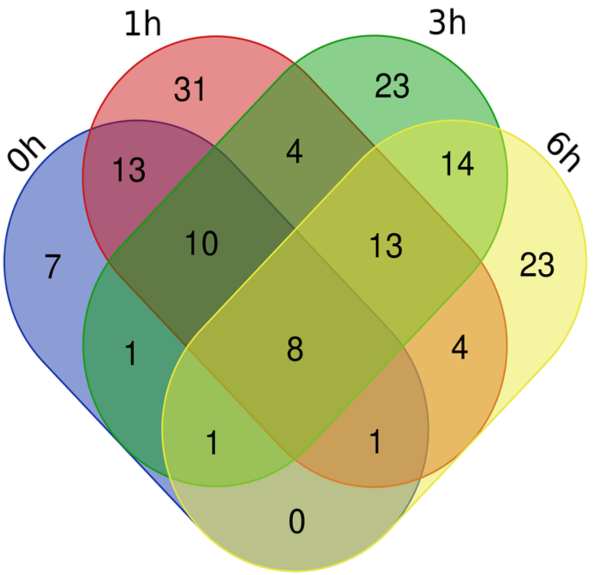

Treatment groups	Total number of genes	Differentially expressed genes
0h 1h 3h 6h	8	*Gm9855, Iigp1, Vcam1, Gbp5, Ctse, Mx1, Gm14513, Ifi202b*
0h 1h 3h	10	*Gm1840, Gbp2, Marco, Saa3, Vsig4, AW112010, Rps15a-ps8, Mrpl48-ps, Il18bp, Rprl3*
0h 1h 6h	1	*Cd69*
0h 3h 6h	1	*Gm5806*
1h 3h 6h	13	*Gm8822, Sele, Gm4955, Prickle1, Gbp3, Ifit2, Gm14446, Fam26f, Cxcl10, Ccl20, Ccl17, Cxcl9, Il12b*
0h 1h	13	*Cd38, Amd2, Aoah, Itgad, Cfb, Ighm, Mmp12, Irg1, Arg2, Cdkn1c, Sncg, Ly6i, Ifi205*
0h 3h	1	*Gm10020*
1h 3h	4	*Ifi44, Gm9770, Rpl15-ps2, Gbp6*
1h 6h	4	*Slfn1, Pydc4, Acp5, Trim30c*
3h 6h	14	*Npl, Fgl2, Tmem132e, Tgtp2, Tex15, Tnfsf9, Nptx1, Diras2, Steap4, Gm1966, Gbp7, Ptges, P2ry13, Vash1*
0h	7	*9430092D12Rik, Adrbk2, Adgb, Col19a1, BC021767, Il1a, Cgn*
1h	31	*Fpr2, Tmprss4, Ovol1, Gm15590, Fpr1, Fosb, Treml4, Gm9115, Treml2, Il23a, Apoc2, Gm15270, F830016B08Rik, Gm21816, Tspan7, Mamdc2, Csf2, Slpi, Hpn, Olfr56, Rasgrp1, Adm, Dio2, Phf11a, Klhl25, Tagap, Gm17709, Six5, Ly6a, Mb21d1, Slfn4*
3h	23	*Mpp7, Igkc, Fam83g, Themis2, I830012O16Rik, Rps15a-ps6, Ifit3, Pilrb1, Slamf9, Pilra, Pcdh10, Gvin1, Ifit1, Fcgr4, Arhgef37, Haao, Cd5l, Gm6969, Slc40a1, Mirlet7c-1, Glns-ps1, Trim34b, Gbp4*
6h	23	*Nos2, BC094916, Gm18445, Ripk2, Gm5431, Il27, Pydc3, Tmem26, Eps8l1, Vipr1, Cd83, Mcemp1, P2ry12, Sparcl1, Prr5l, Ccl12, Tgtp1, Artn, Fam171b, Tnfsf10, Dscaml1, Gm4951, Dnm3*

C

Untreated		1 h post TNF		3h post TNF		6h post TNF	
Gene	Log_2 FC	Gene	Log_2 FC	Gene	Log_2 FC	Gene	Log_2 FC
Ifi202b	12.33919802	*Rprl3*	10.36387211	*Ifi202b*	10.83420325	*Ifi202b*	11.87974009
Gm14513	10.64258095	*Gm14513*	10.00002007	*Rprl3*	10.5563355	*Gm14513*	9.410724294
Rprl3	9.555554473	*Ifi202b*	8.982867338	*Gm14513*	10.09109914	*Gm9855*	4.901651747
Mrpl48-ps	8.155554246	*Mrpl48-ps*	7.800910764	*Rps15a-ps8*	7.380102822	*Gm5806*	4.233735795
Rps15a-ps8	6.790385968	*Rpl15-ps2*	7.712962272	*Rpl15-ps2*	6.803965166	*Ctse*	4.060168512
Gm9855	5.615285295	*Gm1840*	5.174452634	*Glns-ps1*	6.60196019	*Eps8l1*	3.716412273
Gm10020	4.750618992	*Rps15a-ps8*	4.932181935	*Mrpl48-ps*	5.111350853	*Artn*	2.825386825
Sncg	4.220941648	*Sncg*	4.613119352	*Gm5806*	4.921331684	*Gm8822*	2.75666313
Gm5806	4.205630843	*Gm9855*	4.244344963	*Ctse*	4.478020676	*Gm18445*	2.516652736
Gm1840	4.018039654	*Ctse*	4.137432572	*Gm9855*	4.473576621	*Diras2*	2.212689761
Amd2	3.958754696	*Amd2*	4.020767088	***Igkc***	4.176542761	***Ptges***	2.205773632
Ctse	3.563955991	*Gm15270*	3.688591746	*Gm1840*	4.03689499	*Tmem26*	2.007294959
Cdkn1c	2.427557706	*Gm9770*	3.590484222	*Trim34b*	3.889748416	*Npl*	1.962591978
Ighm	1.999212655	*Tspan7*	2.90480288	*Gm10020*	3.699587201	*Mcemp1*	1.690498044
Mmp12	1.533018916	*Cdkn1c*	2.577222529	*Rps15a-ps6*	3.484454392	***Nptx1***	1.595027056
Adrbk2	1.50168423	***Dio2***	2.540602019	*Gm8822*	2.394832319		
		Gm9115	2.533670346	***Nptx1***	2.350311055		
		Gm8822	2.353477053	*Fam83g*	2.311651579		
		Fosb	2.133411628	*Npl*	2.100611669		
		Ighm	2.019931145	*Gm9770*	1.965161379		
		Mamdc2	1.912134511	*Diras2*	1.941471473		
		Gm17709	1.805066879	*Mpp7*	1.601717243		
		Mmp12	1.543076742	***Ptges***	1.561910607		
		Gm15590	1.505256109				

D

Untreated		1 h post TNF		*3h post TNF*		6h post TNF	
Gene	Log_2 FC	Gene	Log_2 FC	Gene	Log_2 FC	Gene	Log_2 FC
Aoah	-1.723942723	***Ifi44***	-1.508991736	***Fcgr4***	-1.500226768	*Gm5431*	-1.508388804
Mx1	-1.74985477	*Treml2*	-1.526851511	*Cd5l*	-1.52801533	***Fam171b***	-1.546525062
Arg2	-1.818682955	*Irg1*	-1.558817021	*Il18bp*	-1.53288762	*Gm4951*	-1.554238882
Adgb	-1.853383991	*Mb21d1*	-1.559622172	*Themis2*	-1.5399538	*Vash1*	-1.571599161
Vcam1	-1.881524004	***Aoah***	-1.574000316	*Gbp7*	-1.564099527	*Tgtp2*	-1.606139259
BC021767	-1.929892569	*Hpn*	-1.578046911	***Ifi44***	-1.600817869	*Ifit2*	-1.61419936
Il18bp	-1.936097825	*Apoc2*	-1.608479323	*AW112010*	-1.609739748	***Nos2***	-1.626198
AW112010	-1.944799971	*Ly6a*	-1.611687538	*Ifit1*	-1.62896429	*Gm14446*	-1.634104469
Gbp2	-2.053246753	*Ifit2*	-1.613924899	*Tgtp2*	-1.647038684	***Ripk2***	-1.638554691
9430092D12Rik	-2.167284487	***Tagap***	-1.641882671	***Mx1***	-1.65903596	*BC094916*	-1.673309225
Cd69	-2.192222092	*Gbp3*	-1.651901168	*Fgl2*	-1.683471917	*Gbp3*	-1.685064192
Iigp1	-2.390509925	*Pydc4*	-1.660079696	*Gbp2*	-1.693125575	*Gm1966*	-1.70153307
Ifi205	-2.39458248	*Arg2*	-1.699376044	*Gvin1*	-1.696999205	*Slfn1*	-1.707909869
Irg1	-2.573435938	*Gm21816*	-1.700475219	***Cxcl10***	-1.6983722	*Acp5*	-1.712839359
Cgn	-2.816376633	*Klhl25*	-1.727028054	*Ifit3*	-1.706277884	***Mx1***	-1.718982094
Itgad	-2.841520673	*Phf11a*	-1.730108094	*Gm1966*	-1.725432901	*Gbp7*	-1.729300164
Col19a1	-2.857668202	*Gm4955*	-1.773551759	*Gbp6*	-1.745991818	*Pydc4*	-1.731152329
Ly6i	-2.929443334	***Adm***	-1.774120237	***Vcam1***	-1.756456851	*Tgtp1*	-1.737611579
Cfb	-2.972620141	*Treml4*	-1.842275494	*I830012O16Rik*	-1.806027342	*Vipr1*	-1.759498063
Vsig4	-3.041467875	*Acp5*	-1.863072285	*Pilra*	-1.826692623	***Cd83***	-1.762320534
Il1a	-3.080985465	***Mx1***	-1.865282133	*Gbp3*	-1.838238382	*Fgl2*	-1.77986668
Marco	-3.116585722	***Slpi***	-1.865941558	***Iigp1***	-1.871040062	*Trim30c*	-1.842660447
Cd38	-3.192987085	*Fam26f*	-1.86835632	*Marco*	-1.899846379	***Vcam1***	-1.843206449
Saa3	-4.277679249	***Iigp1***	-1.871576848	*Pilrb1*	-1.907303026	***Il27***	-1.872914554
Gbp5	-4.454497827	***Cd69***	-1.882494258	*Gm14446*	-1.924517113	***Cd69***	-1.875089623
		Il18bp	-1.903619277	*Tnfsf9*	-1.974511203	*Prr5l*	-1.960758277
		Vcam1	-1.954258707	*Ifit2*	-2.056430652	***Tnfsf10***	-1.994655191
		Six5	-1.955054995	*Prickle1*	-2.157689691	***Ccl20***	-2.022365035
		Csf2	-1.961174798	*Gbp4*	-2.194979358	*Fam26f*	-2.062926995
		Trim30c	-1.963342638	*Vash1*	-2.195799984	***Cxcl10***	-2.129577371
		Ifi205	-1.966541649	*Slc40a1*	-2.23608746	*Gm4955*	-2.166760273
		Sele	-1.967967558	***Ccl20***	-2.268025896	*Dnm3*	-2.173646204
		Cd38	-2.072761173	*Fam26f*	-2.291460625	***Iigp1***	-2.188318038
		Slfn4	-2.133486586	*Saa3*	-2.352178037	*Ccl12*	-2.275811643
		Gbp6	-2.147567398	*P2ry13*	-2.440324937	*Tex15*	-2.278845622
		Fpr1	-2.208627504	*Tmem132e*	-2.484693196	*Pydc3*	-2.362026092
		Gbp2	-2.25017469	*Gm4955*	-2.557796008	*Tmem132e*	-2.373833983
		Prickle1	-2.273664038	*Slamf9*	-2.693271342	***Il12b***	-2.430355083
		Slfn1	-2.372621511	*Tex15*	-2.715103314	*P2ry13*	-2.530071881
		Fpr2	-2.37284256	*Gbp5*	-2.742788056	*Tnfsf9*	-2.554101674
		Gm14446	-2.435600568	***Ccl17***	-2.807017789	***Ccl17***	-2.710687829
		F830016B08Rik	-2.440486439	***Steap4***	-2.961712702	*P2ry12*	-2.720428169
		Rasgrp1	-2.538051405	***Il12b***	-2.974135374	*Dscaml1*	-2.899695934
		Cxcl10	-2.599102579	*Pcdh10*	-3.143113684	*Gbp5*	-3.021606869
		AW112010	-2.7006786	*Mirlet7c-1*	-3.147859928	*Sparcl1*	-3.253807481
		Olfr56	-2.855208751	*Arhgef37*	-3.183746438	***Cxcl9***	-3.313656592
		Ly6i	-2.954106736	*Gm6969*	-3.521442598	***Steap4***	-4.037974196
		Ovol1	-3.042577145	***Sele***	-3.8467232	***Sele***	-4.059546302
		Cfb	-3.095093601	*Vsig4*	-3.846845119	*Prickle1*	-4.303740051
		Ccl20	-3.126021891	***Cxcl9***	-4.026054372		
		Itgad	-3.433148178	*Haao*	-4.094112987		
		Tmprss4	-3.583125889				
		Cxcl9	-3.702313213				
		Vsig4	-3.724019353				
		Il23a	-3.817840079				
		Il12b	-3.968027514				
		Marco	-4.009889267				
		Gbp5	-4.024526427				
		Saa3	-4.23725359				
		Ccl17	-4.358293173				
