# Supplementary material for: Effects of Human RelA Transgene on Murine Macrophage Inflammatory Responses
Source: Biomedicines. 2022 Mar 24;10(4):757. doi: 10.3390/biomedicines10040757 (PMC9027775; doi:10.3390/biomedicines10040757)
Supplement: Supplementary file 1 [file biomedicines-10-00757-s001.zip › Supplementary Materials - Figure S5.pdf]

Effects of human RelA transgene on murine macrophage inflammatory responses. Papoutsopoulou S, Morris L, Bayliff A, Mair T, Stagi M, Bergey F, Alam MT, Sheibani-Tezerji R, Rosenstiel P, Müller W, Martins Dos Santos VAP, Campbell BJ.

SUPPLEMENTARY MATERIALS

Figure S5: Integrative pathway enrichment analysis of differentially expressed genes common to Lipid A and TNF treatment of p65-DsRedxp/IκBα-eGFP BMDMs. Enrichment map and data table indicate the top five prognostic signalling pathways shared by Lipid A-stimulated and TNF-stimulated BMDMs (100 ng/mL Lipid A or TNF 30 ng/mL for 1, 3, and 6 h; N = 3 mice per treatment group); n=61 genes across all treatment groups. Interactions are indicated by connecting lines and multi-coloured nodes indicate pathways that were prognostic based on molecular evidence submitted. Data outputs were generated using the inBio Discover™ tool (www.inbio-discover.com/) and no relevance score cut-off was used.

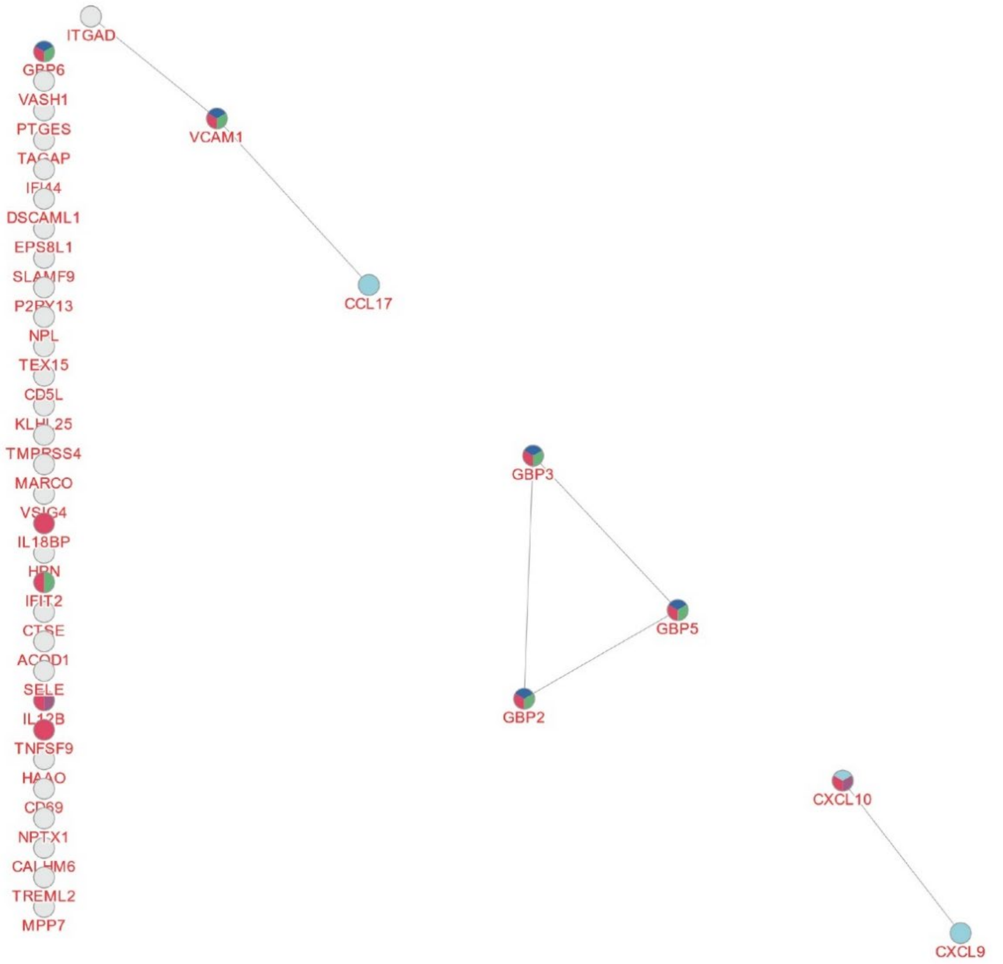

Annotation	Size	Overlap	Ratio	p-Value
Cytokine Signaling in Immune system (R-HSA-1280215)	689	10/38	7.64	4.4e-7
Interferon gamma signaling (R-HSA-877300)	91	5/38	28.92	7.8e-7
Interferon Signaling (R-HSA-913531)	197	6/38	16.03	1.8e-6
Chemokine receptors bind chemokines (R-HSA-380108)	57	3/38	27.70	1.7e-4
Interleukin-10 signaling (R-HSA-6783783)	45	2/38	23.39	3.3e-3
